# Simultaneous Monitoring of Cell-surface Receptor and Tumor-targeted Photodynamic Therapy via TdT-initiated Poly-G-Quadruplexes

**DOI:** 10.1038/s41598-018-23902-5

**Published:** 2018-04-03

**Authors:** Tianhui Shi, Menglin Wang, Hao Li, Miao Wang, Xingyu Luo, Yan Huang, Hong-Hui Wang, Zhou Nie, Shouzhuo Yao

**Affiliations:** State Key Laboratory of Chemo/Biosensing and Chemometrics, College of Biology, College of Chemistry and Chemical Engineering, Hunan University, Changsha, 410082 P. R. China

## Abstract

Cancer cells contain a unique set of cell surface receptors that provide potential targets for tumor theranostics. Here, we propose an efficient approach to construct G-quadruplex-based aptamers that specifically recognize cell-surface receptors and monitor them in an amplified manner. This designed aptamer combined particular sequence for the c-Met on the cell surface and poly-G-quadruplexes structures that allow a rapid and amplified fluorescent readout upon the binding of thioflavin T (ThT). The poly-G-quadruplexes also function as a carrier for photosensitizers such as TMPyP4 in that, the aptamer further trigger the production of reactive oxygen species (ROS) to commit cells to death. This unique c-Met targeting aptamer enabled simultaneous monitoring of c-Met on the cell surface with ThT and photodynamic killing of these lung cancer cells with TMPyP4. This strategy is expected to enhance the development of tumor-targeted diagnosis and drug delivery.

## Introduction

Cell surface receptors play critical roles in physiological and pathological processes including extracellular matrix processing, growth factors signalings, and the activation of cells to microbial invasion^1,2^. Importantly, cell surface receptors are involved in the progression of various degenerative diseases such as cancer, atherosclerosis, and neurological disorder^3^. Therefore, diagnostic targeting and regulation of receptors facilitate the understanding of the major pathological pathways and the development of therapeutic applications^4^.

c-Met is a tyrosine kinase receptor (RTK) for hepatic growth factor (HGF), which plays a significant role in embryonic, neuronal, and muscle development^5^. Dysregulation of HGF/c-Met signaling has been implicated in tumor malignancies through its downstream signaling pathway that mediates proliferation, apoptosis, and migration of cancer cells^6,7^. Given the high correlation with oncogenesis, c-Met is considered as a source of biomarkers for cancer theranostics^8,9^.

A few analyses including western blotting, enzyme-linked immunosorbent assay (ELISA) and flow cytometry are widely used to examine the levels of cell-surface receptors^10–13^. However, these techniques are highly dependent on the qualities of antibodies conjugated with either fluorescent organic dyes or nanoparticles. These methods also require tedious cell fixation and washing steps to achieve sufficient signal to background ratios for cell imaging and analysis. Therefore, they are not cost-effective to monitor cell surface receptors^14^. Besides, monitoring them in live cells remains a major challenge. Thus, biosensing molecules have been incorporated into the cell-surface membrane field and have shown the potential to elucidate cell functions with high spatiotemporal resolution^15^.

Most cell-surface sensors anchor the cell surface with low selectivity, and some fabrication processes require toxic chemical reactions or intrinsic genetic manipulations. Those drawbacks limit the practical usage and further clinical application of some sensors^16–19^. Thus, an approach that allows simple and efficient sensing elements onto the cell membrane without affecting cell physiology would be desirable and highly useful. The establishment of a multifunctional platform may facilitate the monitoring of a variety of cancer biomarkers located on the cell membrane.

As sensing molecules, aptamers have been attractive in the field of cell labeling, cell surface modification, and cell-cell interaction^20–22^. Aptamer binds to target molecules with high affinity and specificity, such as small molecules, proteins, and cells, via its unique secondary or tertiary structures^23,24^. Moreover, aptamers can be applied to a variety of biomedical applications on cell surfaces when combining with other DNA-based reactions and technologies, such as Watson-Crick hybridization, polymerase chain reaction, rolling cycle reaction and DNA-based nanotechnologies^25,26^.

As a therapeutic strategy, photodynamic therapy (PDT) has become a robust platform with specific spatiotemporal selectivity and minimal invasiveness for cancer treatment^27^. PDT usually consists of three components: a photosensitizer, light, and tissue oxygen^28,29^. In a typical PDT for cancer, the light-activated photosensitizer transfers its excited-state energy to the surrounding oxygen for generating reactive oxygen species (ROS), which cause the death of cancerous cells directly or indirectly^30,31^. Since photosensitizers only cause cytotoxicity upon irradiation with the particular types of light, PDT may serve as a “magic bullet” to selectively disrupt malignant tumors, while sparing healthy organs liver, spleen, and kidney^32–35^. Therefore, the development of PDT may bring novel opportunities to future cancer treatment.

**Figure 1:**
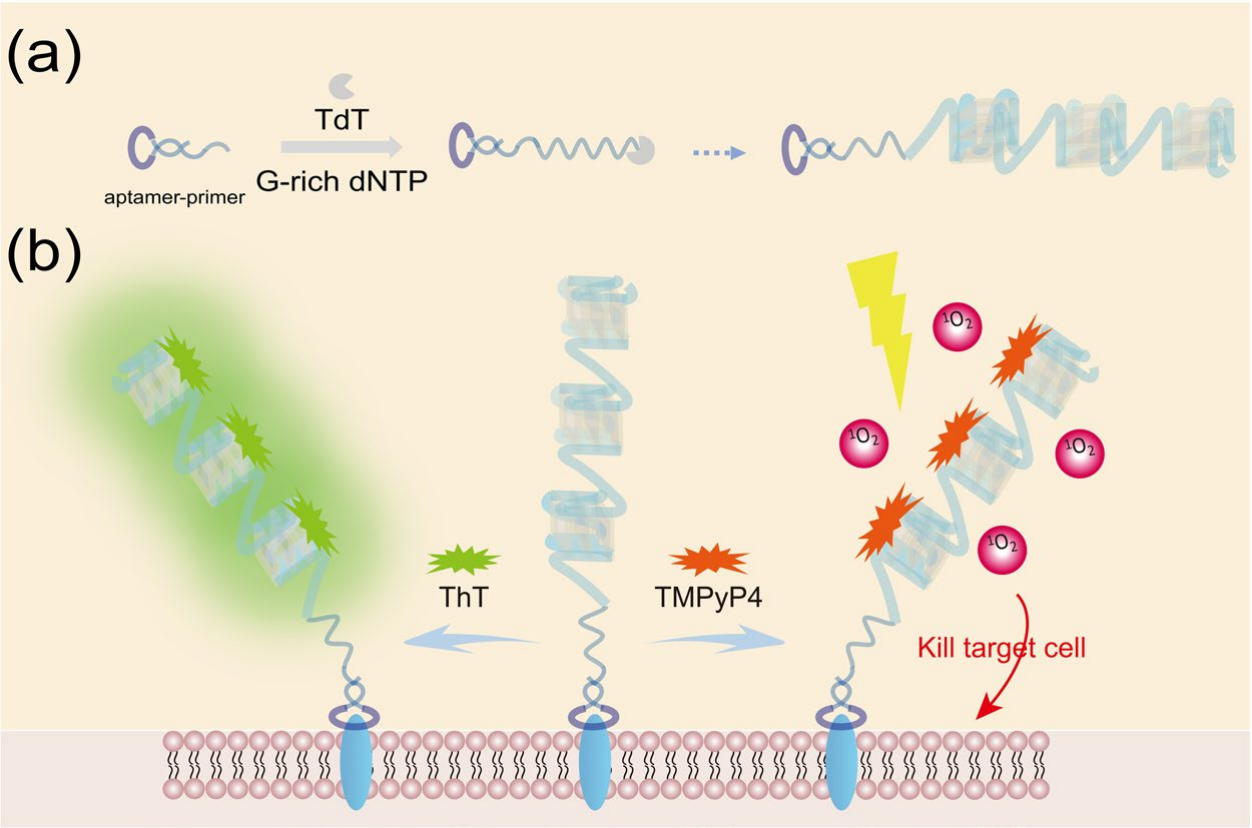
Illustration of TdT initiated poly-G-quadruplexes for simultaneous achieving rapid and amplified monitoring of cell-surface c-Met and cancer-targeted photodynamic therapy.

In this study, we design a simple method for one-step construction of a probe with two functional DNA groups: one is an aptamer group that recognizes the surface receptor of the target cell; the other is a primer group that initiates formation of poly-G-quadruplexes through TdT. As illustrated in Fig. 1, we used of a fluorogenic dye, Thioflavin T, 3,6-dimethyl-2-(4-dimethylaminophenyl) benzthiazolium cation (ThT), for the early detection of amyloid fibrils^36^, the fluorescence signal of ThT is greatly enhanced when binding to G-quadruplex^37^. This strategy allows a sensitive “turn-on” detection mode on target cell surface. Meanwhile, the poly-G-quadruplexes serve as a carrier for photosensitizers with porphyrin molecular structures such as the cationic porphyrin 5, 10, 15, 20-tetra(N-methyl-4-pyridyl) porphyrin (TMPyP4). Because of the recognition function of the aptamer group and the loading function of the poly-G-quadruplexes, the designed probe was delivered to a target cell with high affinity and selectivity. Upon light irradiation, ROS are generated rapidly, and the target cells undergo cell death. Thus, monitoring of receptor on the cell surface and photodynamic killing of the target cancer cells are simultaneously achieved when the probe loaded with both ThT and TMPyP4. Taken together, our study offers not only a promising methodology for tumor-targeted PDT but also a potential strategy for drug delivery with both diagnostic signal and therapeutic effect.

## Results

### Construction of the poly-G-quadruplexes conjugated probe

The primary mechanism is shown in Fig. 1. To construct the poly-G-quadruplexes conjugated probe, we first designed an aptamer-primer with three parts: the 3′-end region with a random primer sequence for *in vitro* TdT polymerization; the 5′-terminal region with an aptamer sequence; a 17 poly-T bases linker between the two regions (Supplemental Fig. 1). The DNA aptamer sequence, SL1, was selected as reported previously^38,39^. That was composed solely of 50-mer single-stranded DNA for binding with the c-Met. To avoid the formation of undesired secondary structures, we used 17 poly-T bases to separate the aptamer sequence and the primer sequence. For *in vitro* elongation reaction, we utilized TdT, a DNA polymerase that catalyzes the addition of deoxynucleotides to the 3′-OH terminal of DNA molecules without the requirement of DNA template^40–42^. In this reaction, the aptamer-primer initiated generation of random arrayed G-rich DNA, forming poly-G-quadruplexes through TdT, which could further conjugate ThT or TMPyP4.

To optimize the performance of this a cell-surface probe, we first evaluated and optimized the appropriate length of the poly-G-quadruplexes by adjusting the concentration of the aptamer-primer and reaction time for TdT polymerization. We found that a longer product around 600 bases could be generated with the increasing concentration of the aptamer-primer within 200 nM range. This observation indicates a close correlation of the product length with the aptamer-primer concentration. Interestingly, a shorter product could be generated with the increasing level of the aptamer-primer above 200 nM range (Supplemental Fig. 2). Thus, we concluded that the elongation product was the longest at 200 nM of the aptamer-primer. We then have optimized the elongation time for the product length with the TdT. It kept increasing till 120 min and reached the plateau afterward (Supplemental Fig. 3). We then set the elongation time for the most extended products as 120 min. Thus, the concentration of aptamer-primer was set at 200 nM and the elongation time was set as 120 min for the optimal length of the product and these conditions were also used in the following experiments.

### Characterization of the poly-G-quadruplexes conjugated probe

**Figure 2:**
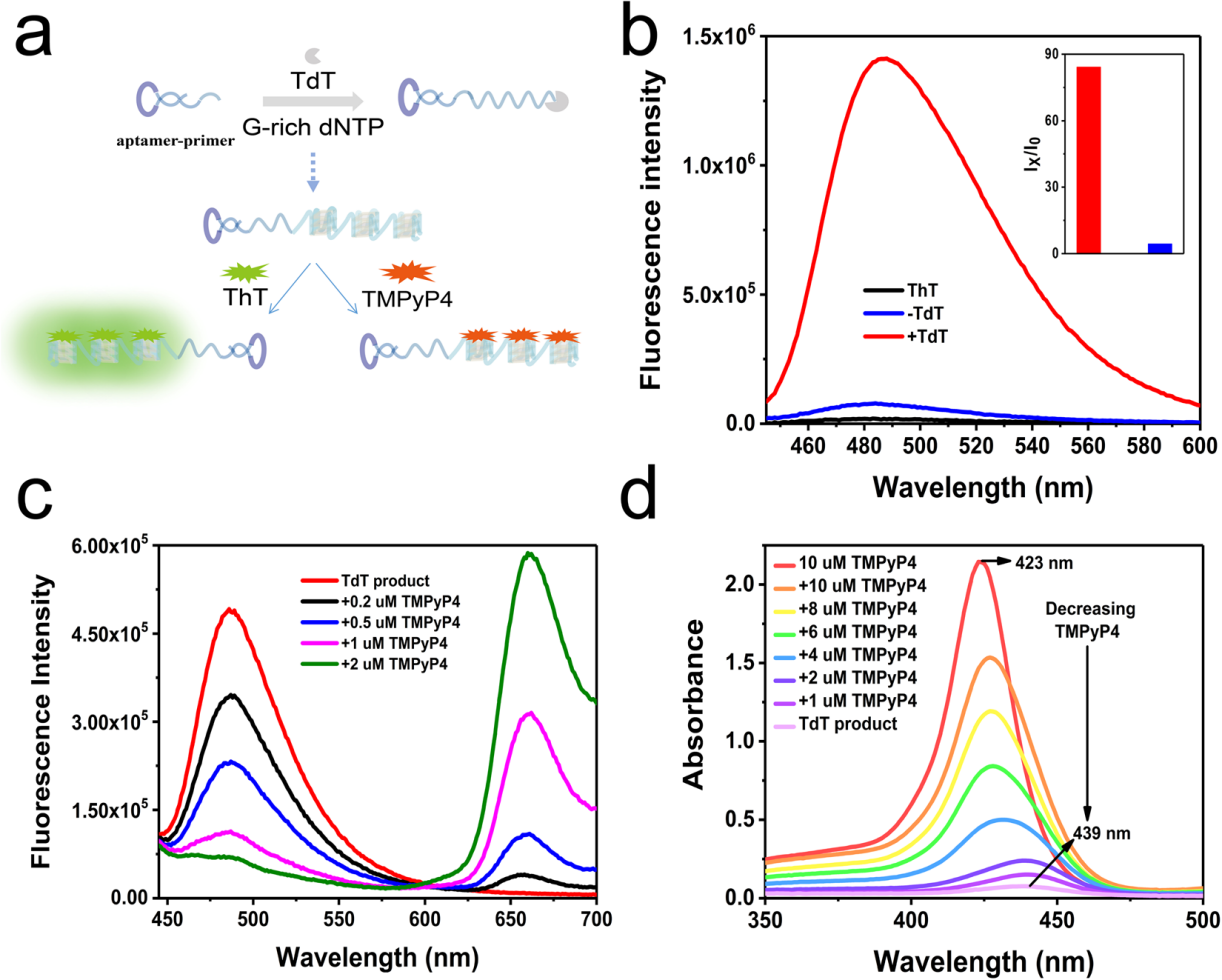
(**a**) Schematic illustration of the poly-G-quadruplexes conjugated probe system. (**b**) Fluorescence emission spectra of ThT in the presence of TdT generated poly-G-quadruplexes. (**c**) Fluorescence emission spectra of the poly-G-quadruplexes-ThT upon addition of the TMPyP4. (**d**) UV-Vis absorption titration spectra of TMPyP4 with the poly-G-quadruplexes.

After we obtained the poly-G-quadruplexes conjugated probe via TdT-initiated G-rich DNA from the aptamer-primer (Fig. 2a), we first characterized the elongation product using the denaturing gel electrophoresis. A strong product band of 120 min polymerization reaction was shown larger than 600 bases (Supplemental Fig. 4a), demonstrating that aptamer-primer was indeed formed a long and stable product as designed. The elongation process was also efficient because only the single band was observed. The global conformation of the elongation product was then determined using circular dichroism (CD) measurements. The CD spectra and the conformations of G-quadruplexes showed an empirical relationship: a positive band at 275 nm and negative band at 250 nm, representing parallel strands; a positive band at 290 nm and a negative band at 260 nm, representing antiparallel strands^43^. An obvious negative peak at 250 nm and a positive peak at 275 nm in the poly-G-quadruplexes conjugated probe indicated that the TdT product indeed formed a parallel G-quadruplex structure (Supplemental Fig. 4b).

We then the binding of the product with ThT (structure shown in Supplemental Fig. 5a) and how the fluorescence signal of ThT was enhanced upon their binding. As shown in Fig. 2b, the product increases ThT fluorescence with a signal-to-background (S/B) ratio of 85 (Fig. 2b inset) after a TdT reaction, while a control experiment was carried out for an amplification reaction without dGTP. The G-excluded TdT product shows no obvious CD signal (Supplemental Fig. 4b) and complete loss of fluorescence in the presence of ThT (Supplemental Fig. 4c), demonstrating that the G-rich sequence is the preliminary condition for poly-G-quadruplex formation. We next monitored changes in fluorescence intensity of ThT at 485 nm over a time course with incubation of poly-G-quadruplexes. The results showed that the fluorescence increases rapidly upon the addition of ThT and saturates fast with several seconds, suggesting that the interaction between ThT and poly-G-quadruplexes happens fast (Supplemental Fig. 6). Since the binding of ThT with the poly-G-quadruplexes is highly selective with strong affinity and rapid kinetics^42^, we reasoned that the poly-G-quadruplexes conjugated probe is adaptable for monitoring cell-surface receptor at spatiotemporal resolution.

We further asked whether the poly-G-quadruplexes conjugated probe could work as the carrier for photosensitizers, advancing its application in PDT. Among photosensitizers, TMPyP4 (structure shown in Supplemental Fig. 5b) is known to bind with a G-quadruplex to stabilize the G-quadruplex formation due to its aromatic and cationic properties^44^. We investigated binding ability between our designed poly-G-quadruplexes and TMPyP4. In a competition assay, the fluorescent intensity of ThT significantly diminished at 485 nm, and the fluorescence intensity of TMPyP4 substantially increased at 660 nm in a dose-dependent manner upon addition of TMPyP4 (Fig. 2c). In a titration of TMPyP4 into free ThT, the fluorescence intensity of TMPyP4 still substantially increased at 660 nm in a dose-dependent manner. However, there was no change in the fluorescent intensity of ThT at 485 nm. (Supplemental Fig. 7a). Then, we found that the emission peak of ThT is at 488 nm, and the excitation peak of TMPyP4 is at 425 nm (Supplemental Fig. 7b). This observation suggests that TMPyP4 acts as a competitive inhibitor for the poly-G-quadruplexes/ThT binding. Meanwhile, the addition of TMPyP4 to poly-G-quadruplexes-ThT complex significantly eliminated the fluorescence signal in solution (Supplemental Fig. 8), supporting the concept that decreased the fluorescence signal comes from the poly-G-quadruplex-TMPyP4 complex. The ultraviolet-visible (UV-Vis) spectrum of TMPyP4 showed an absorption band at 423 nm, which was shifted to 439 nm with the addition of the poly-G-quadruplexes (Fig. 2d). This 16 nm shift was attributed to the binding of TMPyP4 with the poly-G-quadruplexes. The CD spectroscopy result further confirmed the G-quadruplex/TMPyP4 complex conformation. Free TMPyP4 did not show any detectable CD absorption, while the intensities of CD bands accordingly increased after mixing TMPyP4 with a solution of the poly-G-quadruplexes. This observation indicated the formation of a poly-G-quadruplexes-TMPyP4 complex (Supplemental Fig. 9). Therefore, we have developed a poly-G-quadruplexes conjugated probe, which could either quickly respond to ThT for a “light on” detection or stably load TMPyP4 for the potential photodynamic application.

### Cell imaging properties

**Figure 3:**
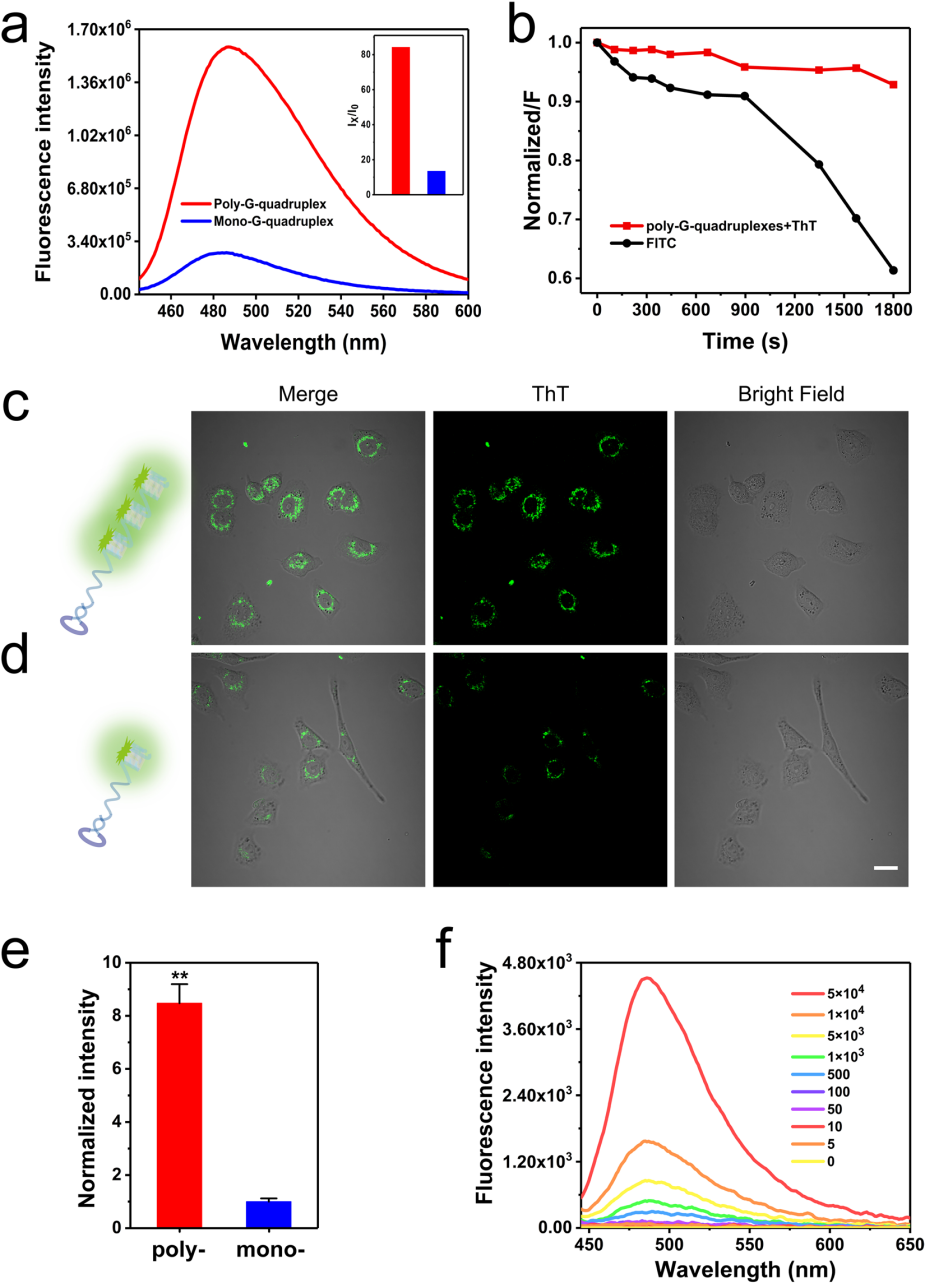
(**a**) Fluorescence emission spectra of ThT in the presence of poly-G-quadruplexes or mono-G-quadruplex. (**b**) Time-dependent photo-bleaching effects of the fluorescent intensity of the poly-G-quadruplexes-ThT complex or FITC, fluorescence intensity was normalized to the maximum intensity of each fluorophore and then plotted against exposure time. Confocal images of A549 cells treated with poly-G-quadruplexes (**c**) and mono-G-quadruplex (**d**). Scale bar: 20 µm. (**e**) The normalized fluorescence intensity of individual cells was quantified from (**c**) and (**d**). Error bars indicate SD, n = 5. **P < 0.01. (**f**) Fluorescence responses of poly-G-quadruplexes-ThT to different numbers of A549 cells.

We also characterized the sensitivity and selectivity of our poly-G-quadruplexes conjugated probe before cell imaging experiments. We confirmed that poly-G-quadruplexes are much more luminescent than mono-G-quadruplex in solution in the presence of ThT (Fig. 3a). Moreover, the poly-G-quadruplexes-ThT complex possesses much better photostability against photobleaching than fluorescein isothiocyanate (FITC), which is a conventional fluorophore employed in cell imaging. The result showed that the fluorescent signal of FITC quickly declined to 61% in 30 min, while the ThT signal shows a negligible decrease over time (Fig. 3b). This excellent anti-photobleaching property probably resulted from that partial exchange of bound ThT with free ThT in solution, which, in turn, prevents the accumulation of photobleached complexes. Such features allow the poly-G-quadruplexes conjugated probe for the use in cell imaging experiments.

The selective binding of the conjugated probe with c-Met was then examined in A549 cells. The Cy5-labeled aptamer-primer led to a more significant shift at both 4 °C and 37 °C as shown by flow cytometric analyses in A549 cells (Supplemental Fig. 10). At the same time, binding to target proteins was confirmed for aptamer-primer and poly-G-quadruplexes by electrophoretic migration shift assays (EMSA; Supplemental Fig. 11). Therefore, the poly-G-quadruplexes probe is expected to recognize c-Met on A549 cell surface. We next utilized poly-G-quadruplexes conjugated probe for cell imaging in the presence of ThT. A549 cells were incubated with the mono- or poly-G-quadruplexes conjugated probe at 4 °C for 30 min, followed by staining with 2 µM ThT and images were acquired on a confocal microscope. The result showed that A549 cells treated with the poly-G-quadruplexes conjugated probe (Fig. 3c) exhibited much stronger fluorescence signal than those treated with the mono-G-quadruplex conjugated probe at the cell membrane (Fig. 3d). Quantification results showed that the fluorescence intensities increased ~8.5 fold in surfaces of A549 cells treated with the poly-G-quadruplexes conjugated probe, compared to those treated with the mono-G-quadruplex probe (Fig. 3e). This result was consistent with fluorescence measurements in solution (Fig. 3a). We further investigated the relationship of fluorescence sensitivity and lengths of poly-G-quadruplexes. We prepared several groups of poly-G-quadruplexes with different lengths by changing the time for the TdT polymerization reaction (Supplemental Fig. 3) and used them for cell imaging. The fluorescence signal of A549 cells gradually increased with increased length of poly-G-quadruplexes. This result can be explained by the fact that longer ploy-G-quadruplexes contain more G-quadruplexes for signal amplification (Supplemental Fig. 12). These results suggested that high fluorescence signal amplification was determined by the quantity of G-quadruplex on the poly-G-quadruplexes conjugated probe. Further, we found that the fluorescence signals are dynamically correlated to the numbers of the target cells within a response range from 0 to 50000 cells (Fig. 3f). The limit of detection is as low as 5 cells because the difference between the fluorescence intensities from 5 cells and control was statistically significant as determined by *t-test* (Supplemental Fig. 13), indicating the advantage of sensitivity enhancement using this poly-G-quadruplexes platform.

### Selective cancer cell targeting

**Figure 4:**
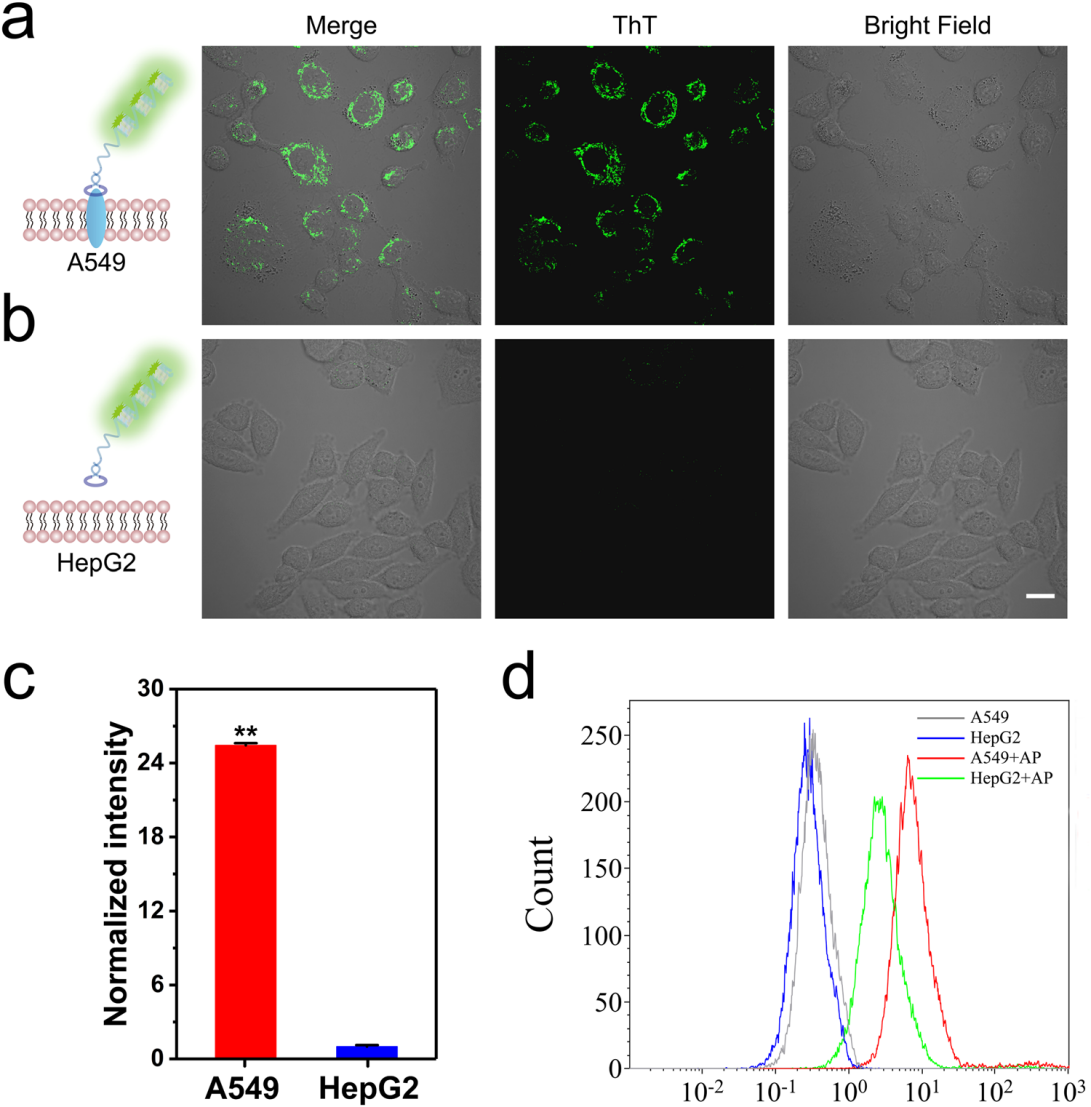
Confocal microscopy images of incubated with poly-G-quadruplexes-ThT probe treated A549 (**a**) and HepG2 (**b**). Scale bar: 20 µm. (**c**) The normalized fluorescence intensity of individual cells was quantified from (**a**) and (**b**). Error bars indicate SD, n = 5. **P < 0.01. (**d**) Flow cytometry analysis of Cy5 labeled aptamer-primer with A549 and HepG2 cells, respectively.

Due to the specific molecular recognition between the aptamer and its target receptor. The probe can selectively anchor on target cell surface, but not on the other cells. To confirm this point, we compared the selective recognition ability of poly-G-quadruplexes conjugated probe between c-Met positive A549 cells and c-Met deficient HepG2 cells. The protein levels of c-Met protein were first measured using an immunoblotting experiment with a specific antibody. For the quantitative analysis, the expression levels of c-Met were normalized to the internal control of tubulin. The near infrared fluorescence signals confirmed the expression of c-Met is much higher in A549 cells than that in HepG2 cells (Supplemental Fig. 14). For cell imaging experiment, both A549 and HepG2 cells were incubated with the probe in the presence of ThT on ice for 30 min, and cell images were acquired on the confocal microscope. We observed bright fluorescence signal from A549 cells surface (Fig. 4a) and little fluorescence signal from HepG2 cells (Fig. 4b). Quantification results also showed that poly-G-quadruplexes conjugated probe efficiently targeted c-Met on A549 cells with ultrahigh signal-to-background (S/B) ratio (Fig. 4c). This result indicated that poly-G-quadruplexes conjugated probe showed significant selectivity for A549 cells, leading to a potential targeted bioimaging ability. The targeting specificity of aptamer-primer toward A549 cells was confirmed by flow cytometry. The Cy5 labeled aptamer-primer showed a stronger binding affinity to A549 cells than the control HepG2 cells at 4 °C (Fig. 4d). These results demonstrated that our poly-G-quadruplexes conjugated probe could target A549 cells with high affinity but HepG2 cells with low affinity. Since a probe for *in vivo* applications should have high biosafety profile, we evaluated the cytotoxicity of the probe using a CCK-8 assay. We observed minimal effects on cell viability at various ThT concentrations up to 10 µM in both A549 and HepG2 cells (Supplemental Fig. 15). The low cytotoxicity of ThT allows for further cell imaging applications for *in vivo* studies. In conclusion, the poly-G-quadruplexes conjugated probe may work as a targeted luminescent probe for bioimaging in live cells.

### Tumor-targeted PDT

**Figure 5:**
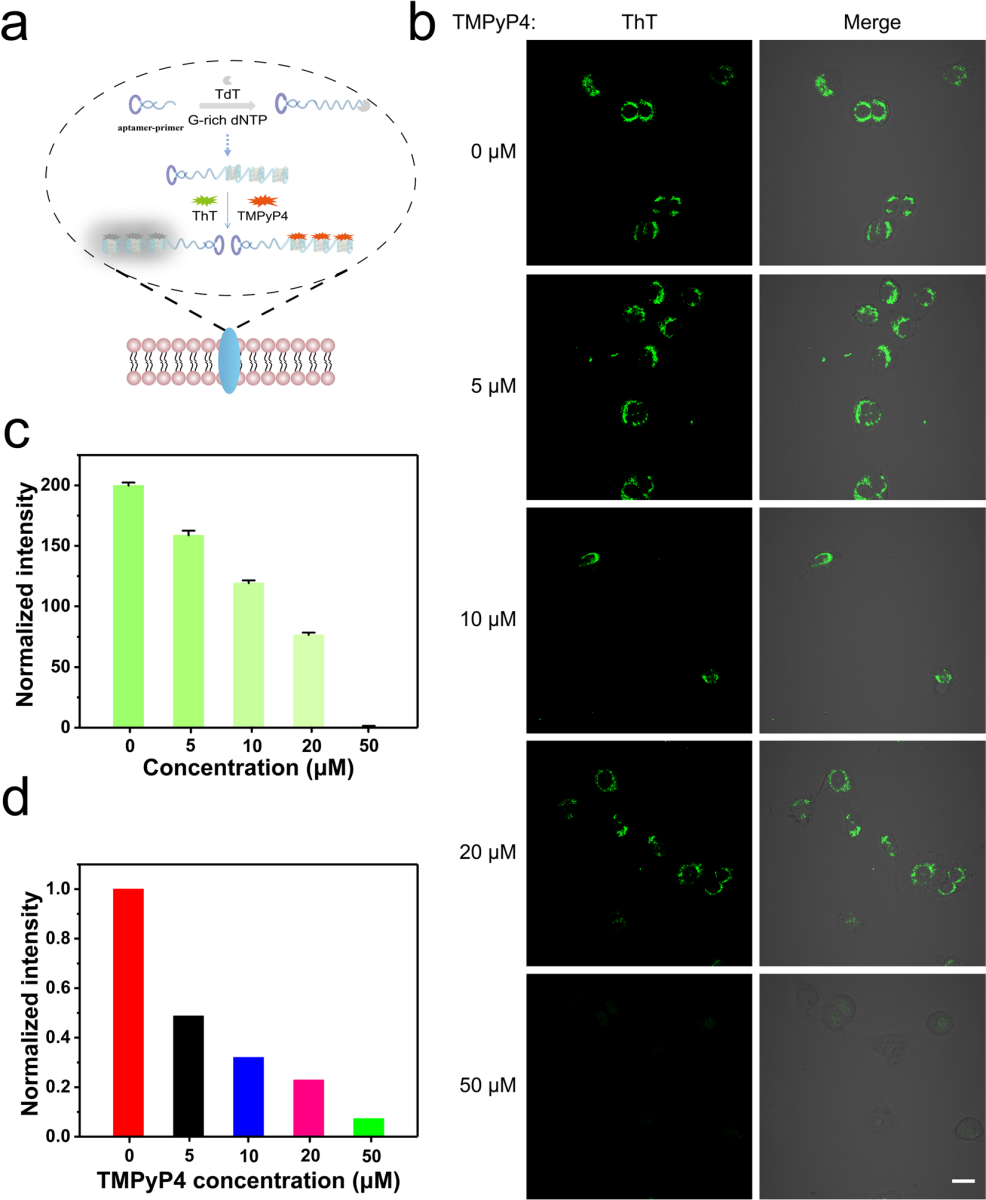
(**a**) Schematic illustration of TMPyP4 competes with ThT in combination with poly-G-quadruplexes on cell-surface. (**b**) Confocal imaging of poly-G-quadruplexes-ThT treated A549 cells with increasing concentrations of TMPyP4 (0, 5, 10, 20, 50 µM). Scale bar: 20 µm. (**c**) The normalized fluorescence intensity of individual cells was quantified from (**b**). Error bars indicate SD, n = 5. (**d**) Cell-surface normalized fluorescence intensity of the ThT at 485nm under increasing concentrations of TMPyP4 (0, 5, 10, 20, 50 µM) by using microplate reader.

PDT is one of the most promising and non-invasive methods for treating malignant or premalignant tissues^27^. TMPyP4 is one of the porphyrin derivatives, and it can be excited by optimal light to generate singlet oxygen in cancer lesions to promote cell death^45^. Target delivery systems for TMPyP4 must increase photosensitizers’ accumulation in the target site and minimize toxicity to the neighboring tissues. We first asked whether the poly-G-quadruplexes conjugated probe can work as both the targeted monitoring and the carrier for TMPyP4 (Fig. 5a). In a competition experiment, we treated A549 cells with the poly-G-quadruplexes conjugated probe in binding buffer (100 µL) on ice for 30 min and then added 200 µL 2 µM ThT with different concentrations of TMPyP4. We observed that the fluorescent signal of ThT at A549 cells membrane significantly diminished upon addition of TMPyP4 (Fig. 5b). Quantification of the confocal images showed that TMPyP4 inhibited the fluorescent signal of ThT in a dose-dependent manner (Fig. 5c), suggesting that TMPyP4 and poly-G-quadruplexes formed a complex. Also, the cell-surface fluorescence intensity of the ThT at 485 nm under different concentrations of TMPyP4 were measured by using microplate reader. Fluorescent signal of ThT at 485 nm decreased with the increased the level of TMPyP4 (Fig. 5d), which is consistent with quantification result from confocal imaging. These results indicated that the TMPyP4 competed with ThT and formed a complex with the poly-G-quadruplexes at the cell surface of A549 cells.

**Figure 6:**
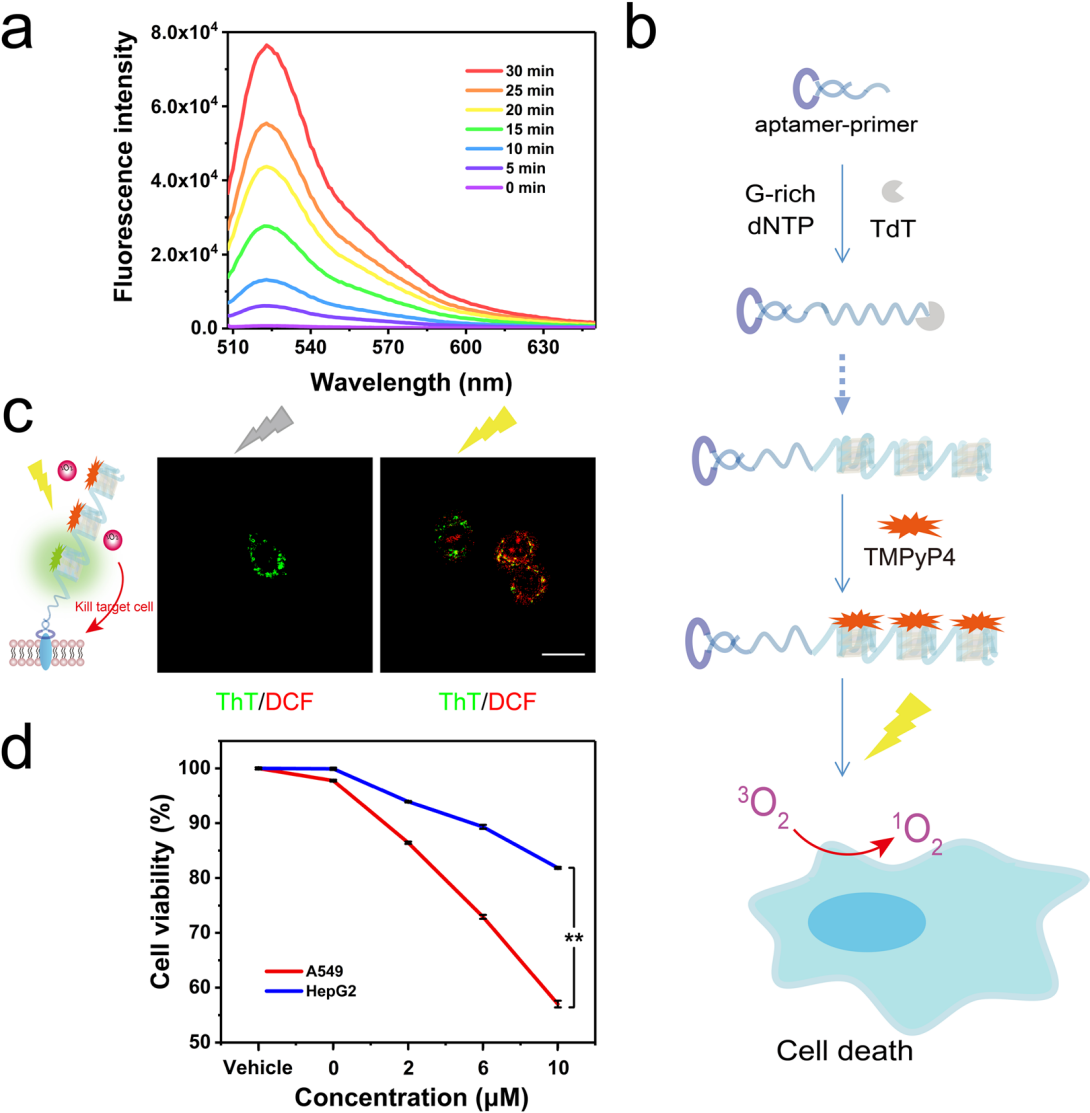
(**a**) Time-dependent ROS generation by poly-G-quadruplexes-TMPyP4 under light irradiation was measured using DCF fluorescence. (**b**) Schematic illustration of the ROS generation from the poly-G-quadruplexes-TMPyP4 system under light irradiation. (**c**) The fluorescence microscopy images of light-induced ROS production of A549 cells treated with poly-G-quadruplexes-ThT-TMPyP4 with or without light irradiation, scale bar: 20 µm. (**d**) Characterization of the selective cytotoxicity of TMPyP4 delivered by the poly-G-quadruplexes conjugated probe to A549 cells (red), HepG2 (blue). Error bars indicate SD, n = 3. **P < 0.01.

We next ask whether targeted delivery of TMPyP4 via the poly-G-quadruplexes conjugated probe exhibited the photodynamic effect to target cells, i.e., A549 cells. To test this, we utilized 2′, 7′-dichlorodihydrofluorescein diacetate (DCFH-DA) to monitor the production of ROS in solution. The result showed that poly-G-quadruplexes-TMPyP4 exhibited a significantly higher ROS production activity under ultraviolet light irradiation (Fig. 6a). A quasilinear correlation was obtained for the peak intensities of poly-G-quadruplexes-TMPyP4 at 525 nm with the light irradiation time (Supplemental Fig. 16). Thus, we hypothesized that poly-G-quadruplexes conjugated probe worked as a carrier of TMPyP4 and selectively anchored on cell-surface receptor via the aptamer group. With light irradiation, poly-G-quadruplexes-TMPyP4 generated ROS and caused photodynamic killing of target cells (Fig. 6b).

Next, we asked whether simultaneous monitoring of cell-surface receptor and tumor-targeted photodynamic effect were possible when poly-G-quadruplexes were loaded with both ThT and TMPyP4 and incubated with A549 cells. Cell imaging data showed that both signals from ThT (green) and the oxidized DCFH-DA (red) are located at the same A549 cells after ultraviolet light irradiation for 10 min (Fig. 6c and Supplemental Fig. 17), suggesting poly-G-quadruplexes-ThT-TMPyP4 has the ability for simultaneous monitoring cell-surface receptor and targeting drug delivery. We next evaluated the photodynamic effect of poly-G-quadruplexes-TMPyP4 on either A549 or HepG2 cells. Cells were incubated with poly-G-quadruplexes, poly-G-quadruplexes-TMPyP4, or free TMPyP4 at 37 °C for 2 h, respectively. Afterward, cells were treated with PDT, or no light irradiation (dark). After treatment, the cell viability after 48 hours’ incubation was determined using the CCK-8 assay (Cell Counting Kit-8, Dojindo, Japan). No obvious change in cell viability was observed for cells treated with poly-G-quadruplexes, poly-G-quadruplexes-TMPyP4, or free TMPyP4 without light irradiation (Supplemental Fig. S18a,b). Under light irradiation (PDT), the results showed the poly-G-quadruplexes-TMPyP4 caused higher phototoxicity than free TMPyP4 in both cells (Supplemental Fig. S18c), suggesting poly-G-quadruplexes conjugated probe increased the photodynamic effect of TMPyP4. Moreover, the poly-G-quadruplexes-TMPyP4 treatment significantly reduced the viability of A549 cells compared to HepG2 cells in a dose-dependent manner (Fig. 6d), indicating that the poly-G-quadruplexes-TMPyP4 can selectively kill c-Met overexpressing A549 cells. These results implied the potential of the poly-G-quadruplexes conjugated probe for therapeutic applications by drug targeted delivery.

## Discussion

Cell-surface receptors play critical roles in physiological and pathological processes. Meanwhile, tumor-targeted photodynamic therapy holds great promise for improving the therapeutic index and reducing side effects. In conventional methodologies, the ability to simultaneous monitor expressions of cell-surface receptor and deliver reagents of bioactivity in living cells remains a major challenge. In this study, we have constructed a new class of poly-G-quadruplexes conjugated probe that can be used for simultaneous monitoring of cancer cell surface receptor and tumor-targeted photodynamic therapy. The poly-G-quadruplexes carrier showed potential impacts on diverse aspects. First, this self-assembly method through enzymatic reaction formulating a carrier was easily performed compared to the chemically covalent synthetic method. Second, the randomly arrayed poly-G-quadruplexes can be combined with the fluorescent dye to enhance the fluorescence signal without chemical modification. Thus, it has the advantages of low cost, high specific/non-specific signal ratio and stability. Moreover, poly-G-quadruplexes hold great potential for several advanced bioimaging techniques, including near-infrared or two-photon imaging, through incorporation with different probes. Third, the whole process avoids the time-consuming light protection steps such as incubation with antibodies and washing. Last, the carrier showed drug loading capacity and targeted drug delivery that improves therapeutic efficacy and reduces side effects of drugs. Therefore, our poly-G-quadruplexes conjugated probe provides the potential for a highly efficient nanocarrier for targeted delivery.

## Methods

### TdT-mediated elongation

The typical TdT-mediated elongation experiment was performed in 10 µL of TdT buffer (1×, 0.2 M potassium cacodylate, 25 mM Tris-HCl, 0.01% (v/v) Triton X-100, 1 mM CoCl_2_, pH 7.2) containing the ssDNA oligo, 1 mM dNTP (10% dTTP, 40% dATP and 50% dGTP), and 4 U of TdT at 37 °C for 2 h, and terminated by heating the solution at 75 °C for 10 min. For TdT-mediated elongation was performed in 10 µL of TdT buffer containing 200 nM aptamer-primer, 1 mM dNTP (10% dTTP, 40% dATP and 50% dGTP), and 4 U of TdT to initiate the elongation at 37 °C for 2 h and terminated by heating the solution at 75 °C for 10 min.

### Fluorescence measurements

Ten microliter TdT-mediated elongation samples were mixed with 90 µL reaction solution containing 50 µL G-quadruplex dyes buffer (2×, 100 mM Tris-HCl, 100 mM KCl, pH 7.4), 38 µL ultrapure water, and 2 µL 100 µM G-quadruplex specific dyes, ThT, and the total volume was 100 µL. Fluorescence experiments were carried out using a QuantaMaster^TM^ fluorescence spectrophotometer (PTI, Canada). ThT was excited at 425 nm, and its emission was recorded from 445 to 600 nm with the maximum emission wavelength at 485 nm. All experiments were performed at least three times.

### UV absorption titration

The titration was performed with a Beckman DU-800 spectrophotometer. The concentration of poly-G-quadruplexes was held constant. Different concentrations (0, 0.5, 1, 1.5, 2, 2.5, 3, 4, 5, 6, 8 µM) of TMPyP4 in buffer versus poly-G-quadruplexes were tested.

### Cell culture

A549 cells (human lung adenocarcinoma epithelial cell line) and HepG2 cells (human hepatocellular carcinoma cell line) were cultured in high glucose Dulbecco’s Modified Eagle’s Medium (DMEM, Sigma-Aldrich, St. Louis, MO) with 10% fetal bovine serum (FBS) and 0.5 mg/mL penicillin-streptomycin in a humidified incubator at 37 °C under a 5% CO_2_ atmosphere. Cells were washed before and after incubation with washing buffer [4.5 g/L glucose and 5 mM MgCl_2_ in Dulbecco’s PBS with calcium chloride and magnesium chloride (Sigma-Aldrich)]. Binding buffer used for selection was prepared by adding yeast tRNA (0.1 mg/mL; Sigma-Aldrich) and BSA (1 mg/mL; Fisher Scientific) to the wash buffer to reduce background binding.

### Binding ability

Flow cytometry was used to evaluate the binding ability of aptamer-primer conjugates toward specific cells. Briefly, cells were firstly harvested with 0.02% EDTA to prepare cell suspensions. 1 × 10^5^ cells were incubated with 200 nM aptamer-primer in 100 µL binding buffer at 4 °C or 37 °C for 30 min. Aptamer-primer was labeled with Cy5. After incubation, the cells were washed twice times with 200 µL of washing buffer, and 20000 counts cells were suspended in binding buffer (200 µL) before flow cytometry analysis (BD Biosciences, Mountain View, CA, USA).

### Imaging sensitivity

For confocal imaging, the cells were seeded at 35 mm confocal dish in complete medium for 24 h incubation at 5% CO_2_ and 37 °C. A549 cells were first washed with washing buffer at 4 °C, and then incubated with poly-G-quadruplexes (200 nM) or mono-G-quadruplex (200 nM) in binding buffer (100 µL) on ice for 30 min. Subsequently, the cells were washed twice with washing buffer, and then added 200 µL 2 µM ThT. Live cell imaging was performed under confocal laser scanning microscope (CLSM) (Nikon, Eclipse TE2000-E) with a 60× oil immersion objective (Olympus, Melville, NY). Excitation wavelength and emission filters: ThT Blue channel: excitation 405 nm, emission bandpass (430–460 nm) filter. To quantify the fluorescence intensity generated on the cell surface, images were analyzed by imageJ software following the standard guide and references.

Confocal imaging was also used to evaluate the imaging sensitivity of poly-G-quadruplexes with different lengths in binding buffer at 4 °C. The experimental details were similar to the above procedures.

Samples with varying A549 cell numbers ranging from 0 to 5 × 10^4^ in 100 µL binding buffer were obtained by serial dilution.

### Photo-bleaching analysis

1 µM ThT and FITC solution were respectively added to the 10 µL TdT enzymatic reaction system on the glass-bottom dishes, and circular glass slides were used to keep the solution stagnant. Photo-bleaching experiments were performed on the Nikon confocal microscope with 100 mW solid-state laser for 30 min. Fluorescent images were captured every 8 s with the camera through the 10× objective lens and analyzed with the ROI analysis of NIS-element viewer. Fluorescence intensity was normalized to the maximum intensity of each fluorophore and then plotted against exposure time.

### Selective recognition ability

For confocal imaging, the cells were seeded at 35 mm confocal dish in complete medium for 24 h incubation at 5% CO_2_, and 37 °C. A549 or HepG2 cells were first washed with washing buffer at 4 °C and then incubated with poly-G-quadruplexes (200 nM) in binding buffer (100 µL) on ice for 30 min. Subsequently, the cells were washed twice with washing buffer, and then added 200 µL 2 µM ThT. Live cell imaging was performed under confocal laser scanning microscope (CLSM) (Nikon, Eclipse TE2000-E) with a 60× oil immersion objective (Olympus, Melville, NY). Excitation wavelength and emission filters: ThT Blue channel: excitation 405 nm, emission bandpass (430–460 nm) filter. To quantify the fluorescence intensity generated on the cell surface, images were analyzed by image J software following the standard guide and references.

Flow cytometry was also used to evaluate the selective recognition ability of poly-G-quadruplexes in binding buffer at 4 °C. The experimental details were similar to the above procedures.

### G-quadruplex ligand competition

Firstly, confocal imaging was used to evaluate the competition ability of TMPyP4. The cells were seeded at 35 mm confocal dish in complete medium for 24 h incubation at 5% CO_2_ and 37 °C. A549 cells were first washed with washing buffer at 4 °C and then incubated with poly-G-quadruplexes (200 nM) in binding buffer (100 µL) on ice for 30 min. Subsequently, the cells were washed twice with washing buffer and then added 200 µL 2 µM ThT with different concentrations of TMPyP4. Live cell imaging was performed under confocal laser scanning microscope (CLSM) (Nikon, Eclipse TE2000-E) with a 60× oil immersion objective (Olympus, Melville, NY). Excitation wavelength and emission filters: ThT Blue channel: excitation 405 nm, emission bandpass (430–460 nm) filter. To quantify the fluorescence intensity generated on the cell surface, images were analyzed by imageJ software following the standard guide and references.

Microplate reader was used to evaluate the competition ability of TMPyP4. The experimental details were similar to the above procedures.

Fluorescence measurements of G-quadruplex ligand competition, firstly, the poly-G-quadruplexes and ThT staining were performed as the typical procedures described in the experimental section. Then, G-quadruplex ligand TMPyP4 (0–8 µM) was added to the solution to compete with the ThT (2 µM) at 37 °C for 10 min. The fluorescence measurement was carried out using the emission scan mode of the QuantaMaster^TM^ fluorescence spectrophotometer, PTI (Canada). The excitation and the emission wavelengths for ThT were 425 nm and 485 nm respectively. All experiments were performed at least three times.

### Intracellular ROS measurement

Intracellular ROS generation was measured by using 2′, 7′-dichlorofluorescin diacetate (DCFH-DA). DCFH-DA was hydrolyzed enzymatically by intracellular esterases to non-fluorescent DCFH, which remained trapped within the cells. DCFH could react with the intracellular ROS to generate a fluorescent compound dichlorofluorescein (DCF). DCF had excitation/emission maxima of 488 nm/525 nm enabling detection. After various treatments, the cells were loaded with DCFH-DA in DMEM for 30 min, followed by washing several times and exposure under light irradiation for 10 min, and intracellular ROS generation was evaluated using microplate reader and fluorescence microscopy.

### Cytotoxicity test

Cell toxicity was tested by measuring the cell viability by CCK-8 assay, after treatment with poly-G-quadruplexes-TMPyP4 or TMPyP4 alone and irradiation. Cells irradiated by the same lamp without drug treatment were considered to be 100% viable. In brief, A549 and HepG2 cells were cultured at a density of 5 × 10^4^ cells per well (in 100 mL fresh medium) in flat-bottomed 96-well plates. Then TMPyP4 only, poly-G-quadruplexes, or poly-G-quadruplexes-TMPyP4 in 100 µL of binding buffer was added to the respective test well. The cells were incubated at 37 °C in a 5% CO_2_ atmosphere for 2 hours. Then, the supernatant was removed from the test well, and 100 µL of fresh cell culture medium was added and exposure under light irradiation for 10 min. After another 48 h of incubation at 37 °C in a 5% CO_2_ atmosphere, a standard CCK-8 assay followed, CCK-8 assay reagent was added to each well according to the manufacturer’s instructions. After 1–4 h in culture, the cell viability was determined by measuring the absorbance at 450 nm using a microplate reader.

### Statistics

Statistical significance was determined by Student’s t-test or one-way ANOVA followed by Student-Newman-Keuls test using Sigma Stat version 3.1. P < 0.01 was considered statistically significant.

## Electronic supplementary material

Supplementary Information

## References

[1] Mager MD, LaPointe V, Stevens MM (2011). Exploring and exploiting chemistry at the cell surface. Nat Chem.

[2] Ali MM (2012). Cell-surface sensors: lighting the cellular environment. Wiley Interdiscip Rev.

[3] Christopoulos A (2002). Allosteric binding sites on cell-surface receptors: novel targets for drug discovery. Nat Rev Drug Discov.

[4] Johnsson K (2009). Visualizing biochemical activities in living cells. Nat Chem Biol.

[5] Ueki R, Atsuta S, Ueki A, Sando S (2017). Nongenetic Reprogramming of the Ligand Specificity of Growth Factor Receptors by Bispecific DNA Aptamers. J Am Chem Soc.

[6] Gherardi E, Birchmeier W, Birchmeier C (2012). Targeting MET in cancer: rationale and progress. Nat Rev Cancer.

[7] Van Der Steen N (2015). cMET in NSCLC: Can We Cut off the Head of the Hydra? From the Pathway to the Resistance. Cancers.

[8] Boccaccio C, Comoglio PM (2006). Invasive growth: a MET-driven genetic programme for cancer and stem cells. Nat Rev Cancer.

[9] Birchmeier C, Birchmeier W, Gherardi E, Vande Woude GF (2003). Met, metastasis, motility and more. Nat Rev Mol Cell Biol.

[10] Trusolino L, Bertotti A, Comoglio PM (2010). MET signalling: principles and functions in development, organ regeneration and cancer. Nat Rev Mol Cell Biol.

[11] Ducrest AL, Amacker M, Lingner J, Nabholz M (2002). Detection of promoter activity by flow cytometric analysis of GFP reporter expression. Nucleic Acids Res.

[12] Baskar S (2008). Unique cell surface expression of receptor tyrosine kinase ROR1 in human B-cell chronic lymphocytic leukemia. Clin Cancer Res.

[13] Kaur J, Bachhawat AK (2009). A modified Western blot protocol for enhanced sensitivity in the detection of a membrane protein. Anal Biochem.

[14] Odell ID, Cook DJ (2013). Immunofluorescence techniques. J Invest Dermatol.

[15] Jiang F (2015). Progress and Challenges in Developing Aptamer-Functionalized Targeted Drug Delivery Systems. Int J Mol Sci.

[16] Zhao W (2011). Cell-surface sensors for real-time probing of cellular environments. Nat Nanotechnol.

[17] Tokunaga T (2012). Cell surface-anchored fluorescent aptamer sensor enables imaging of chemical transmitter dynamics. J Am Chem Soc.

[18] Giepmans BN, Adams SR, Ellisman MH, Tsien RY (2006). The fluorescent toolbox for assessing protein location and function. Science.

[19] Beigi R, Kobatake E, Aizawa M, Dubyak GR (1999). Detection of local ATP release from activated platelets using cell surface-attached firefly luciferase. Am J Physiol.

[20] Zhu GZ (2013). Building fluorescent DNA nanodevices on target living cell surfaces. Angew Chem Int Ed.

[21] Xiong X (2013). DNA aptamer-mediated cell targeting. Angew Chem Int Ed.

[22] Tan W, Donovan MJ, Jiang J (2013). Aptamers from cell-based selection for bioanalytical applications. Chem Rev.

[23] Szeitner Z, Lautner G, Nagy SK, Gyurcsanyi RE, Meszaros T (2014). A rational approach for generating cardiac troponin I selective Spiegelmers. Chem Commun.

[24] Liang H (2014). Functional DNA-containing nanomaterials: cellular applications in biosensing, imaging, and targeted therapy. Acc Chem Res.

[25] Wu C (2015). A survey of advancements in nucleic acid-based logic gates and computing for applications in biotechnology and biomedicine. Chem Commun.

[26] Cansiz S (2015). DNA Aptamer Based Nanodrugs: Molecular Engineering for Efficiency. Chem Asian J.

[27] Fan W, Huang P, Chen X (2016). Overcoming the Achilles’ heel of photodynamic therapy. Chem Soc Rev.

[28] Wang C, Cheng L, Liu Z (2013). Upconversion nanoparticles for photodynamic therapy and other cancer therapeutics. Theranostics.

[29] Agostinis P (2011). Photodynamic therapy of cancer: an update. Ca-Cancer J Clin.

[30] Karunakaran SC (2013). *In vitro* demonstration of apoptosis mediated photodynamic activity and NIR nucleus imaging through a novel porphyrin. ACS Chem Biol.

[31] Liu K (2012). Covalently assembled NIR nanoplatform for simultaneous fluorescence imaging and photodynamic therapy of cancer cells. ACS Nano.

[32] Ethirajan M, Chen Y, Joshi P, Pandey RK (2011). The role of porphyrin chemistry in tumor imaging and photodynamic therapy. Chem Soc Rev.

[33] Huang P (2011). Photosensitizer-conjugated magnetic nanoparticles for *in vivo* simultaneous magnetofluorescent imaging and targeting therapy. Biomaterials.

[34] Huang P (2011). Folic Acid-conjugated Graphene Oxide loaded with Photosensitizers for Targeting Photodynamic Therapy. Theranostics.

[35] Huang P (2012). Light-triggered theranostics based on photosensitizer-conjugated carbon dots for simultaneous enhanced-fluorescence imaging and photodynamic therapy. Adv Mater.

[36] Khurana R (2005). Mechanism of thioflavin T binding to amyloid fibrils. J Struct Boil.

[37] Mohanty J (2013). Thioflavin T as an efficient inducer and selective fluorescent sensor for the human telomeric G-quadruplex DNA. J Am Chem Soc.

[38] Ueki R, Sando S (2014). A DNA aptamer to c-Met inhibits cancer cell migration. Chem Commun.

[39] Ueki R, Ueki A, Kanda N, Sando S (2016). Oligonucleotide-Based Mimetics of Hepatocyte Growth Factor. Angew Chem Int Ed.

[40] Leung KH (2015). Development of an Aptamer-Based Sensing Platform for Metal Ions, Proteins, and Small Molecules through Terminal Deoxynucleotidyl Transferase Induced G-Quadruplex Formation. ACS Appl Mater Interfaces.

[41] Liu Z (2014). Randomly arrayed G-quadruplexes for label-free and real-time assay of enzyme activity. Chem Commun.

[42] Liu Z (2017). Enzyme-Activated G-Quadruplex Synthesis for *in Situ* Label-Free Detection and Bioimaging of Cell Apoptosis. Anal Chem.

[43] Vorlickova M (2012). Circular dichroism and guanine quadruplexes. Methods.

[44] Shieh YA, Yang SJ, Wei MF, Shieh MJ (2010). Aptamer-based tumor-targeted drug delivery for photodynamic therapy. ACS Nano.

[45] Yuan Q (2013). Targeted bioimaging and photodynamic therapy nanoplatform using an aptamer-guided G-quadruplex DNA carrier and near-infrared light. Angew Chem Int Ed.

