# Supplementary material for: Simultaneous Monitoring of Cell-surface Receptor and Tumor-targeted Photodynamic Therapy via TdT-initiated Poly-G-Quadruplexes
Source: Sci Rep. 2018 Apr 3;8:5551. doi: 10.1038/s41598-018-23902-5 (PMC5882647; doi:10.1038/s41598-018-23902-5)
Supplement: Supplementary file 1 — Supplementary Information [file 41598_2018_23902_MOESM1_ESM.docx]

**Supplementary Information**

**Simultaneous Monitoring of Cell-surface Receptor and Tumor-targeted Photodynamic Therapy via TdT-initiated Poly-G-Quadruplexes**

Tianhui Shi, Menglin Wang, Hao Li, Miao Wang, Xingyu Luo, Yan Huang, Hong-Hui Wang^*^, Zhou Nie^*^, Shouzhuo Yao

State Key Laboratory of Chemo/Biosensing and Chemometrics, College of Biology, College of Chemistry and Chemical Engineering, Hunan University, Changsha, 410082, P. R. China.

^*^E-mail address: wanghonghui@hnu.edu.cn (H. H. W.); niezhou.hnu@gmail.com (Z. N.); Tel.: +86-731-88821626; Fax: +86-731-88821848

**Contents**

[**Experimental Section** 3](#_Toc450577026)

Optimized design for in vitro experiment [3](#_Toc450577028)

Denaturing 8% PAGE [3](#_Toc450577031)

Circular dichroism experiments  [3](#_Toc450577031)

Electrophoretic Mobility Shift Assay  [3](#_Toc450577031)

Near-infrared DyLight680/800 western blot analysis [4](#_Toc450577031)

Biocompatibility assay [4](#_Toc450577031)

[**Figures** 5](#_Toc450577034)

[S1 5](#_Toc450577035)

[S2 6](#_Toc450577036)

[S3 7](#_Toc450577037)

[S4 8](#_Toc450577037)

[S5 9](#_Toc450577037)

[S6 10](#_Toc450577038)

[S7 11](#_Toc450577039)

[S8 12](#_Toc450577039)

[S9 13](#_Toc450577039)

[S10 14](#_Toc450577039)

[S11 15](#_Toc450577039)

[S12 16](#_Toc450577039)

[S13 17](#_Toc450577039)

[S14 18](#_Toc450577039)

[S15 19](#_Toc450577039)

[S16 20](#_Toc450577039)

[S17 21](#_Toc450577039)

[S18 22](#_Toc450577039)

**Experimental Section**

**Optimized design for in vitro experiment**

For concentration, the reaction mixture consisted of 1 mM dNTP monomers, 20 nM, 50 nM, 100 nM, 200 nM, 500 nM, or 1000 nM aptamer-primer, and 4 U of TdT in 10 µL of TdT buffer was incubated at 37 ºC for 2 h and the reaction was then stopped by heating at 75 ºC for 10 min. For time, the reaction mixture consisted of 1 mM dNTP monomers, 200 nM aptamer-primer, and 4 U of TdT in 10 µL of TdT buffer was incubated at 37 ºC for 0 min, 30 min, 60 min, 90 min, 120 min, and 180 min, respectively, and the reaction was then stopped by heating at 75 ºC for 10 min.

**Denaturing 8% PAGE**

For the denaturing 8% PAGE analysis of the TdT polymerization product, TdT polymerization process was conducted as the same as aforementioned. After TdT reaction, the sample was loaded into a denaturing 8% PAGE, and the electrophoresis was carried in 1× Tris-borate-EDTA (TBE) buffer (89 mM tris(hydroxymethyl)aminomethane, 2 mM ethylenediaminetetraacetic acid and 89 mM boric acid, pH 8.0) at 110 V for 3 h. The gel was stained by SYBR Green II for 30 min and scanned by a ChemiDoc™ MP System (Bio-Rad).

**Circular dichroism experiments**

The **C**D spectra were recorded on MOS-500 spectrophotomer (BioLogic, France) at room temperature. Before the CD measurement, 100 µL of the TdT-elongated aptamer-primer (200 nM) samples were mixed with 100 µL Tris buffer (100 mM Tris-HCl, 100 mM KCl, pH 7.4) for 30 min at room temperature. The mixture was transferred to 2 mm path length quartz cuvettes for measurement, scanning from 210 to 350 nm at a rate of 50 nm/min for three times and keeping the slit to 5 nm. Each spectrum was corrected by subtracting the CD data of the Tris buffer alone in the same quartz cell.

**Electrophoretic Mobility Shift Assay**

200 nmol of Aptamer-primer (AP), Lib-primer (LP), and their TdT extension products were incubated with 200 nmol of BSA or c-Met-Fc in 10 μL of DPBS for 30 min at 37 °C. 2 μl of 6× DNA gel loading buffer were added, and the total volume was run on a water-cooled 4-20% Tris-borate-EDTA gel followed by SYBR Green II and detection with a ChemiDoc™ MP System (Bio-Rad).

**Near-infrared DyLight680/800** **western blot analysis**

The western blot experiments were based on the protocols provided by Abcam Company. In brief, A549 and HepG2 cells were grown for 24 hours as described on 35 mm culture plates until 80-100% confluent. Total cellular protein was harvested utilizing CelLytic™ cell lysis Reagent (Sigma-Aldrich Co.) and complete protease inhibitors (Roche Biochemicals), then centrifuged at 14000 g for 10 min at 4 °C to discard cellular debris, finally, the supernatant was collected. 20 µL of total protein for each sample were mixed with 5× loading buffer and heated at 98 °C for 5 min. The proteins were analyzed by 8% SDS-PAGE with 1× SDS running buffer and then transferred to 0.22 µm NC membrane (10 min). Then the membrane was blocked with BSA (5% PBST: PBS+5% Tween-20) for 1 h and incubated with primary antibody (rabbit anti-Met, 1:1000 dilution) at 4 °C overnight. After that, the membrane was washed 3 times with PBST for 5 min, then incubated with secondary antibody DyLight800 conjugated goat anti-rabbit IgG (Thermo Fisher, 1:2500 dilution) for 1h at RT and washed 3 times with PBST. The samples were detected with near-infrared laser imaging scanning system (Odyssey Clx). After that, another 20 µL (the same amount as the above) of each sample was analyzed in 8% SDS-PAGE and transferred to 0.22 µm NC membrane (10 min). The membrane was incubated with mouse anti-a tubulin antibody (1:2500 dilution) for 1h at RT, washed 3 times with PBST, then incubated with DyLight680 conjugated goat anti-mouse secondary antibody (Thermo Fisher, 1:2500 dilution).

**Biocompatibility assay**

The CCK-8 assay was performed as a preliminary screening for the evaluation of cytotoxicity of ThT for each type of cell. Briefly, a sample of A549 or HepG2 cells were seeded at 1×10^4^ cells per well into 96-well plates for 24 h. Various concentrations of ThT ( 0, 0.5, 1, 2, 4, 6, 8 and 10 µM) were added, and the cells were further incubated for 24 h, in a humidified incubator at 37 °C containing CO_2_ (5%). To evaluate the cell viability, the medium was removed, and then 10 µL of CCK-8 solution diluted in fresh medium (100 µL) was added to each well and incubated at 37 °C for 1-4 h. After this, the absorbance value at 450 nm was recorded using a microplate reader. Each concentration was tested at least three times. Cell viability was calculated as described by the manufacturer.

**
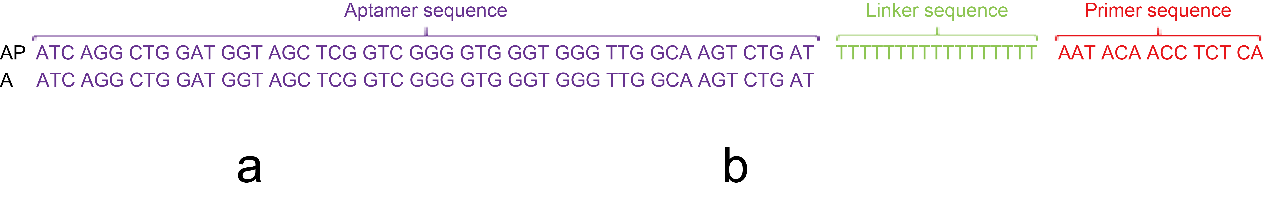
**

**Figure S1.** Sequence information and schematic representation of the aptamer used in the main text (a) aptamer-primer (AP) and (b) aptamer (A). Seventeen nucleotide bases (green letters in the sequence) was inserted between the primer sequence (red) and aptamer sequence (purple) as a “flexible linker” to confer flexibility.


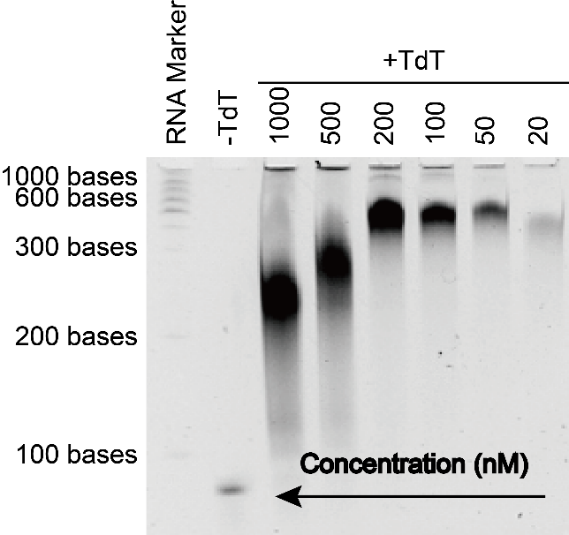


**Figure S2.** Denaturing 8% PAGE analysis of the poly-G-quadruplexes synthesized by TdT with various concentration of aptamer-primer, from 20 to 1000 nM. The TdT reaction mixture contained 4 U TdT, and 1 mM dNTP and the polymerization reaction lasted for 120 min.


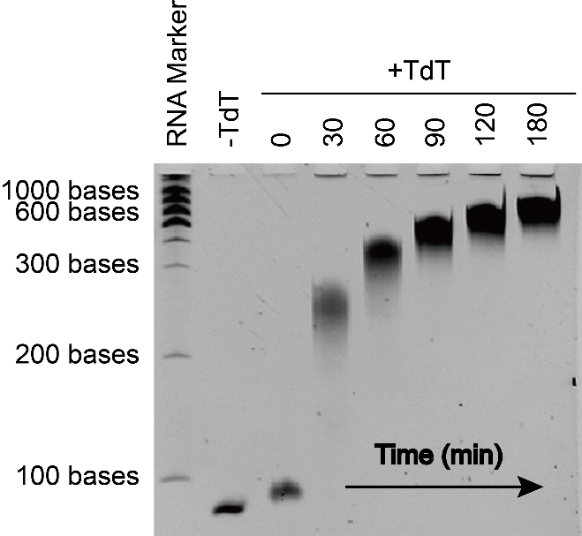


**Figure S3.** Denaturing 8% PAGE analysis of the poly-G-quadruplexes synthesized by TdT with various time points, from 0 to 180 min. The TdT polymerization was performed with 200 nM aptamer-primer, 4 U TdT, and 1 mM dNTP, then lasted for different formation times.


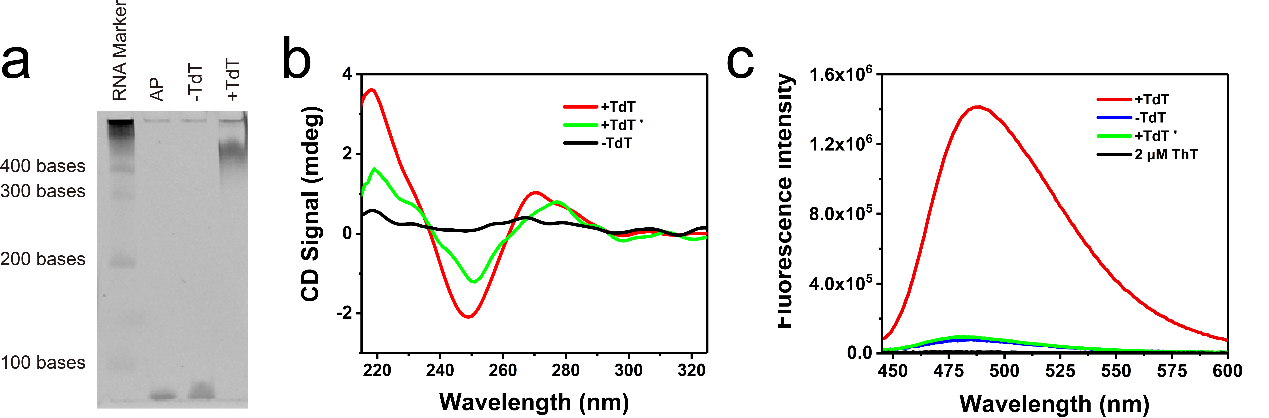


**Figure S4.** (a) Polyacrylamide gel electrophoresis: RNA marker (lane 1), aptamer-primer (lane 2), reaction mixture without TdT (lane 3) and with TdT (lane 4). (b) CD spectra analysis. Black curve: aptamer-primer, red curve: TdT polymerization products (dNTP pool: 10%dTTP, 40% dATP and 50% dGTP), green curve: TdT polymerization product (dNTP pool: 50% dTTP and 50% dATP. (c) Fluorescence emission spectra of ThT. Red curve: ThT and TdT polymerization product (dNTP pool: 10%dTTP, 40% dATP and 50% dGTP), blue curve: ThT and aptamer-primer (without TdT), green curve: ThT and TdT polymerization product (dNTP pool: 50% dTTP and 50% dATP), black curve: ThT.


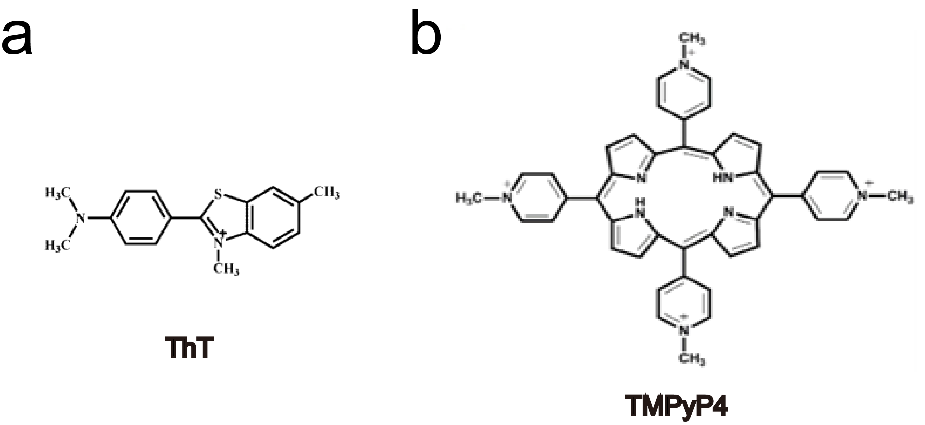


**Figure S5.** The molecular structures of ThT (a) and TMPyP4 (b). The common point of their molecular structure is that all of them are cationic compounds, and the nitrogen atoms of the nitrogen methyl in the molecule all have a positive charge.


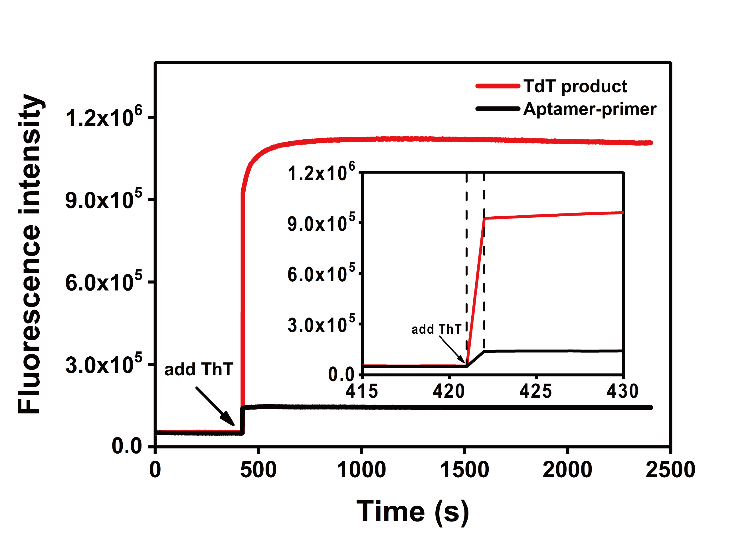


**Figure S6.** Kinetic study. Real-time recording of the fluorescence emission intensity changes as a function of time upon addition of ThT to the TdT-generated poly-G-quadruplexes. The TdT-generated poly-G-quadruplexes was prepared in the reaction mixture containing 200 nM DNA aptamer-primer, 4 U TdT, and 1 mM dNTP. The final concentration of ThT was 2 µM. The total volume of the sample was 100 µL. The excitation wavelength was fixed at 425 nm, and the emission wavelength of ThT is 485 nm, respectively.


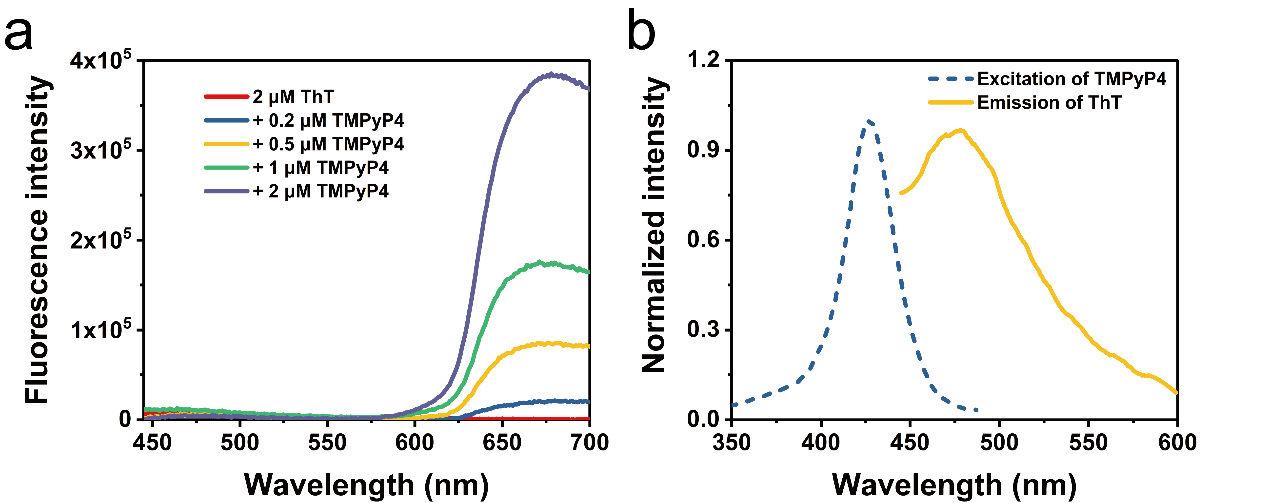


**Figure S7.** (a) Fluorescence emission spectra of a titration of TMPyP4 into free ThT (2 μM). (b) Fluorescence emission spectra of ThT (2 μM) and fluorescence excitation spectra of TMPyP4 (2 μM). The intensity has been normalized.


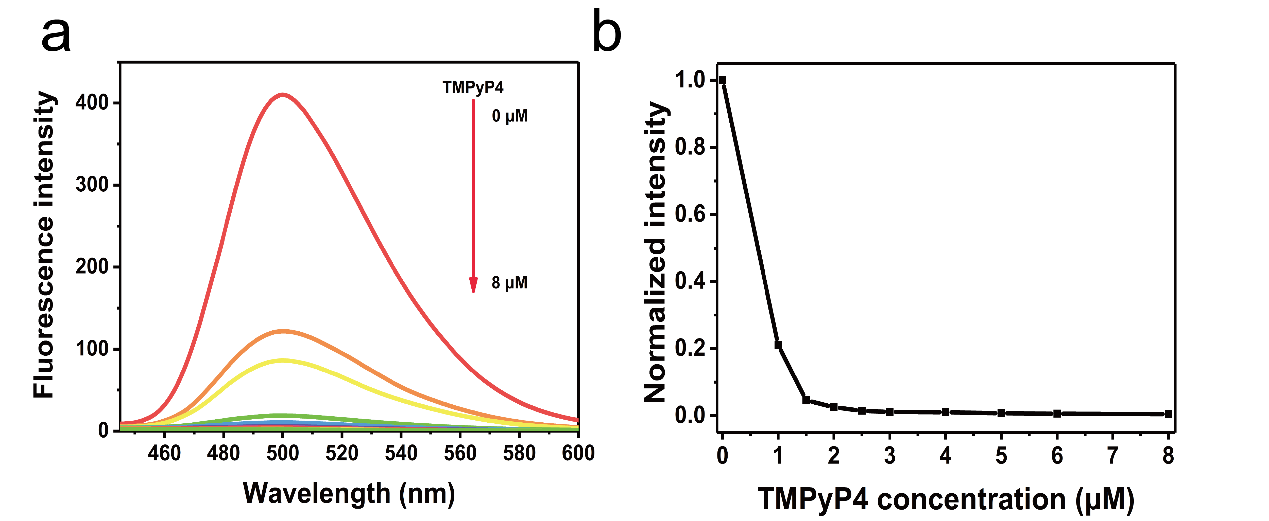


**Figure S8.** (a) Fluorescence emission spectra of the poly-G-quadruplexes-ThT upon addition of the TMPyP4. (b) Normalized fluorescence intensities of ThT (2 µM) at 485 nm in the presence of poly-G-quadruplexes were plot as a function of different concentrations of TMPyP4 equivalents (0-8 µM).


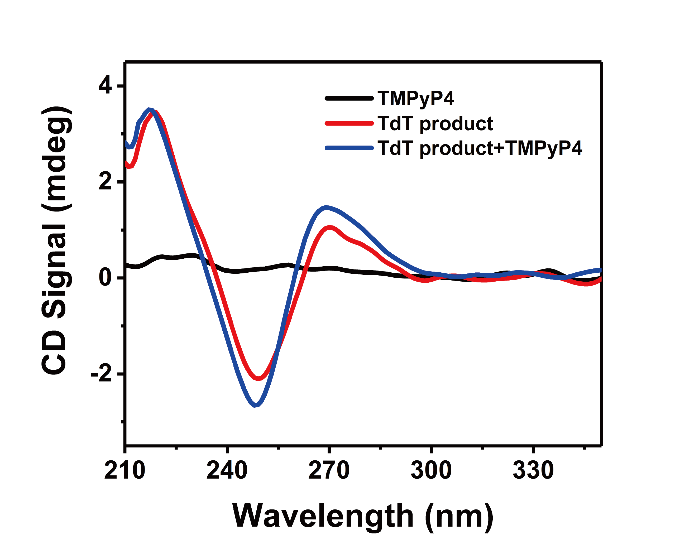


**Figure S9.** CD spectra of TMPyP4 (black curve), the TdT-initiated poly-G-quadruplexes (red curve), and the TdT-initiated poly-G-quadruplexes-TMPyP4 (blue curve).


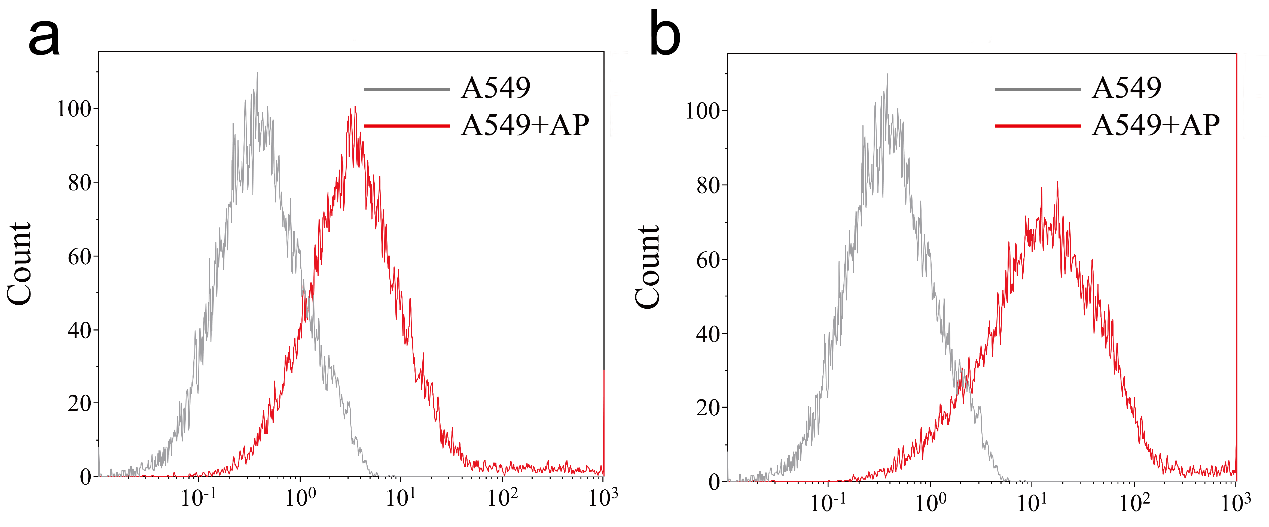


**Figure S10.** Flow cytometric analysis of 3′-Cy5 labeled aptamer-primer (200 nM) binding to A549 cells at 4 °C (a) or 37 °C (b). DNA sequence was incubated with cells in binding buffer at 4 °C or 37 °C for 30 min. After incubation, the cells were washed with washing buffer twice and then analyzed by flow cytometry.

| AP | ATCAGGCTGGATGGTAGCTCGGTCGGGGTGGGTGGGTTGGCAAGTCTGATTTTTTTTTTTTTTTTTTAATACAACCTCTCA |
| --- | --- |
| LP | ATTTAGAGTGACGCAGCAGGACACGGTGGCTTAGTTTTTCAAGGCTTAGTTTTTTTTTTTTTTTTTTAATACAACCTCTCA |


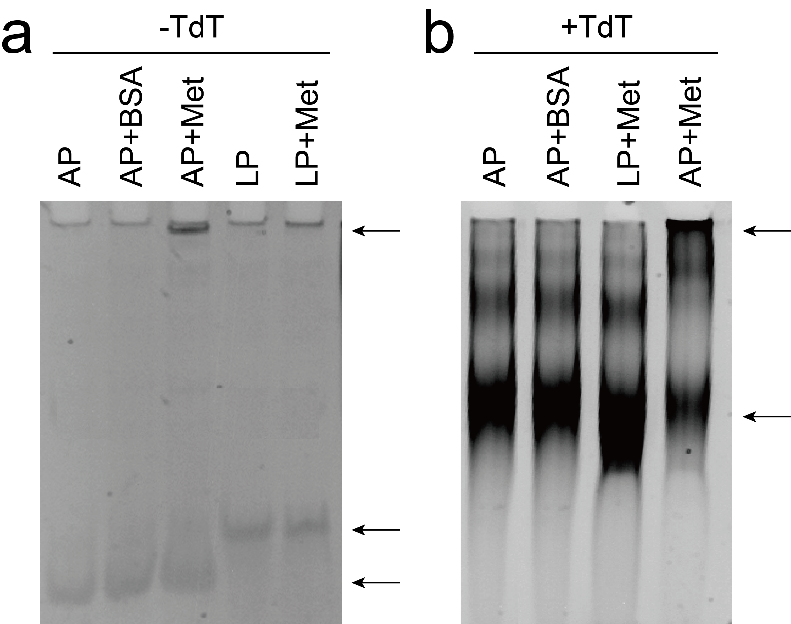


**Figure S11.** Naturing PAGE analysis of the binding of AP (a) or poly-G-quadruplexes (b) to c-Met. The TdT reaction mixture contained 4 U TdT, and 1 mM dNTP and the polymerization reaction lasted for 120 min. AP (additional band in lane 3, left), and poly-G-quadruplexes (additional band in lane 4, right) exhibited binding to c-Met-Fc fusion proteins. Negative control BSA, LP, and its extended product did not show a migration shift, as expected.


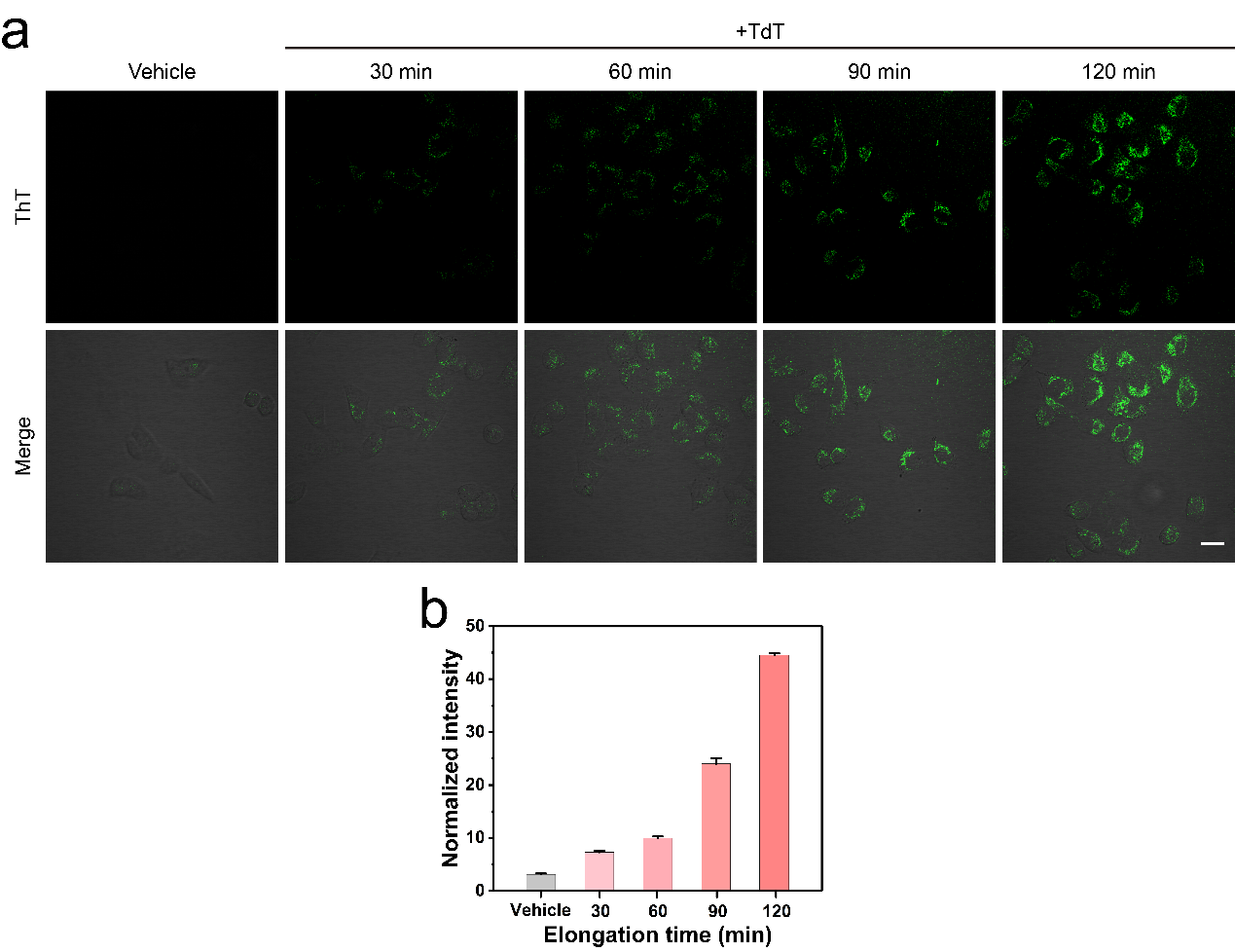


**Figure S12.** (a) Poly-G-quadruplexes with different lengths for A549 cells imaging by confocal microscopy. The cells were incubated at 4 °C for 30 min. Scale bar: 20 μm. (b) Normalized fluorescence intensity of individual cells was quantified from (a). Error bars indicate SD, n=4.


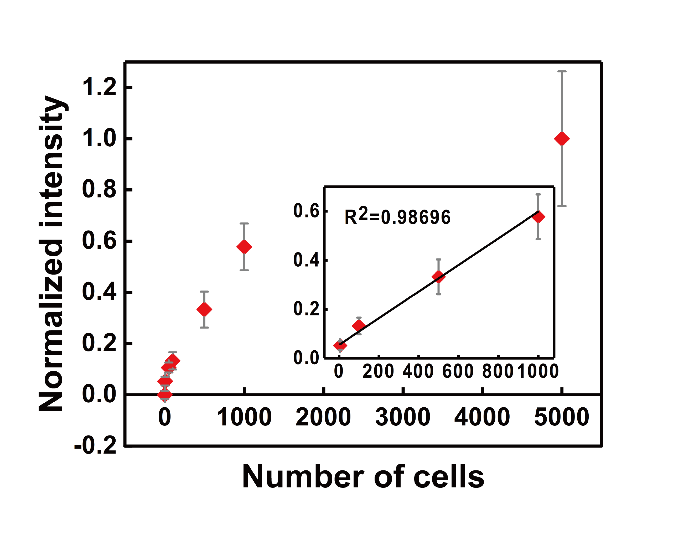


**Figure S13.** The plot of normalized fluorescence intensity at 485 nm versus the number of the cell for A549 cells. Error bars indicate SD, n=3.


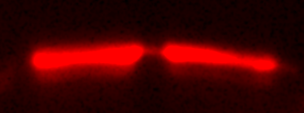

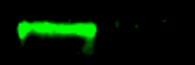


Tubulin

c-Met

55

140

**A549**

**HepG2**

kD

**Figure S14.** Analysis of total c-Met expression by Near-infrared DyLight680/800 western blot in A549 and HepG2 cells. Tubulin was included as an internal control.


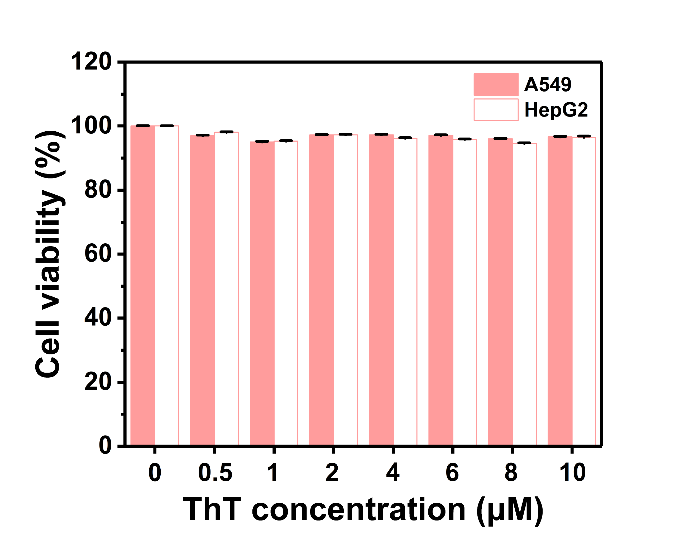


**Figure S15.** (a) Cytotoxicity of ThT with A549 and HepG2 cells. The cells were incubated with ThT at various concentrations (0.5, 1, 2, 4, 6, 8, 10 µM) for 24 hours at 37 °C containing CO_2_ (5%) and the cell viability was determined using CCK-8 assay. The cells incubated without ThT was used as a control. Error bars indicate SD, n=6.


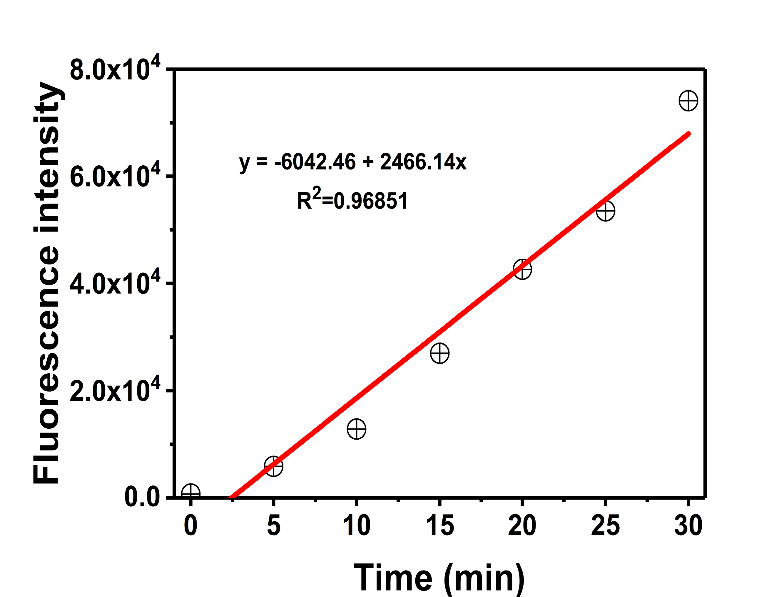


**Figure S16.** Fluorescence intensities of DCF at 525 nm in the presence of poly-G-quadruplexes-TMPyP4 were plot as a function of different irradiation time.


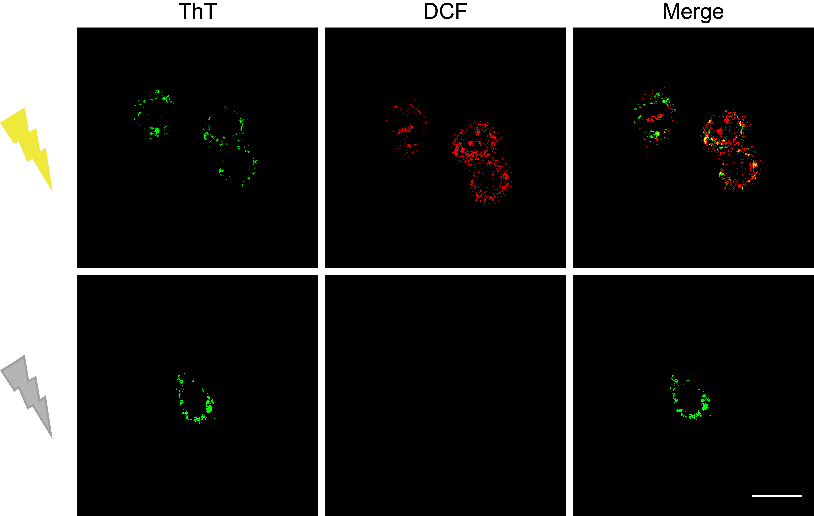


**Figure S17.** The fluorescence microscopy images of light irradiation induced ROS production of A549 cells treated with poly-G-quadruplexes-ThT-TMPyP4. Scale bar: 20 µm.


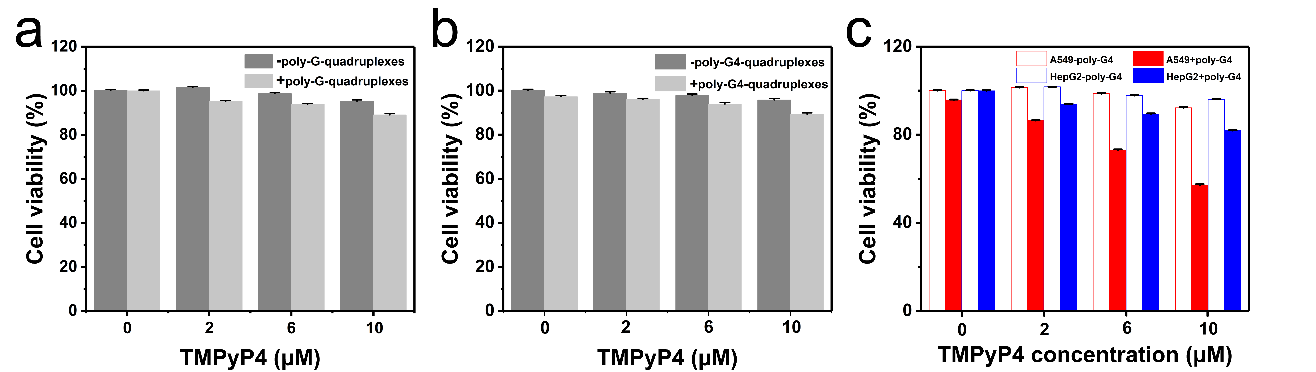


**Figure S18.** The dark toxicity of the poly-G-quadruplexes with and without the TMPyP4 in A549 cells (a) and HepG2 cells (b). (c) Characterization of the selective cytotoxicity of TMPyP4 delivered by poly-G-quadruplexes. A549 cells (red) and HepG2 cells (blue) were incubated with poly-G-quadruplexes-TMPyP4 (right bar) or free TMPyP4 (left bar) followed by light irradiation for 10 min. The 48-hour cell viability was determined using the CCK-8 assay. Error bars indicate SD, n=3.
